# Chemical characterization and discovery of novel quality markers in *Citrus aurantium* L. fruit from traditional cultivation areas in China using GC–MS-based cuticular waxes analysis

**DOI:** 10.1016/j.fochx.2023.100890

**Published:** 2023-09-20

**Authors:** Yan Li, Jie Liu, Jie Li, Haijing Xiao, Yiyun Xu, Siqing Fan, Zhaoqi Xie, Min Guo, Jiaxin Yang, Xue Jing, Chunsong Cheng

**Affiliations:** a Lushan Botanical Garden, Chinese Academy of Science, Jiujiang City, Jiangxi Province, PR China; b School of Food and Pharmaceutical Engineering, Zhaoqing University, Zhaoqing City, Guangdong Province, PR China; c Faculty of Chinese Medicine, Macau University of Science and Technology, Macao, PR China; d School of Environmental and Chemical Engineering, Zhaoqing University, Zhaoqing City, Guangdong Province, PR China; e Jiujiang Academy of Agricultural Sciences, Jiujiang City, Jiangxi Province, PR China; f National Resource Center for Chinese Materia Medica, Chinese Academy of Chinese Medical Sciences, Beijing, PR China

**Keywords:** *Citrus aurantium* L., Fruit, Cuticular wax, Fatty acid, GC–MS, Linoleic acid (PubChem CID: 5280450), Stearic acid (PubChem CID: 5281), Tangeretin (PubChem CID: 68077), Nobiletin (PubChem CID: 72344).

## Abstract

*Citrus aurantium* L. fruit is a commonly used Chinese medicine whose therapeutic effects tends to be affected by growing conditions. In order to gain insights into the effects of growing location on the cuticular wax composition of *C. aurantium* L. fruit, we analyzed the differences in the wax composition of its fruits collected from different regions. The findings showed that the cuticular waxes in the fruit peels were mainly composed of fatty acids, which differed quantitatively in the chemical profiles of *C. aurantium* L. samples from different geographical conditions. Particularly, the concentrations of linoleic acid and stearic acid in the total component content of the fruit peel were above 1%, with a greater level in the geo-authentic samples. Thus, GC–MS-based wax analysis was first used for the chemical characterization and quantification of cuticular waxes, which could be considered as a rapid way for evaluating the quality of medicinal fruits.

## Highlights

• The cuticular waxes in the peel of *Citrus aurantium* L. fruit are mainly composed of fatty acids.
• The high concentrations of linoleic acid and stearic acid are associated with *Citrus aurantium* L. fruits collected from geo-authentic areas.
• The high presence of fatty acids is considered to contribute principally to the identification of *Citrus aurantium* L. fruits from different growing locations.

## 1 Introduction

*Citrus aurantium* L. (*C. aurantium* L.), also known as sour orange or bitter orange, is a member of the genus *Citrus* belonging to the Rutaceae family. The genus *Citrus* is recognized to have originated in the tropical and subtropical regions of Southeast Asia, where China is an important center of origin for this genus (Jing et al., 2015). The varieties of *C. aurantium* L. include *C. aurantium* L. cv. *Huangpi-Suanchen* (Chinese name ‘Choucheng’), *C. aurantium* L. var. *amara* Engl (Chinese name ‘Daidai’), *C. aurantium* L. var. *decumana* Bonar (Chinese name ‘Zhuluan’), and *C. aurantium* L. cv. *Tangcheng* (Gao et al., 2020). The tree of *C. aurantium* L. is widely cultivated in China, especially in Jiangxi, Hunan, Sichuan, and Zhejiang Provinces. The fruits, leaves, flowers, or peels of *C. aurantium* L. are widely used not only for the preparation of food by-products, such as marmalades, flavorings, and perfumes, but also used for medicinal purposes to treat gastrointestinal disturbances, to assist with mild insomnia, to provide skin care, to reduce blood glucose, and to prevent viral diseases (De Santis and Frangipane, 2020, Degirmenci and Erkurt, 2020, Farag et al., 2020). It has been revealed that the methanol extract of the dried immature peel of *C. aurantium* L. showed anticancer properties (Han et al., 2012). The fruit of *C. aurantium* L. is a source of health-giving essential oils that can be applied as antioxidant and bacteriostatic agents (Oulebsir et al., 2022, Tundis et al., 2012).

All terrestrial plants are covered with a hydrophobic layer called cuticle, which is a protective barrier against environmental stresses. Wax is one of the components in the cuticle (Chai et al., 2020, Chu et al., 2017). Previous reports showed that the basic components of cuticular waxes in plants were very-long-chain aliphatic components (chain length from C12 up to C70) including alcohols, alkenes, aldehydes, ketones, fatty acids, and esters (Klavins and Klavins, 2020, Shepherd and Wynne Griffiths, 2006). Cuticular wax compositions have an influence on the quality of fruits during storage and their shelf life. The chemical composition of cuticular waxes varies in plant species and organs, as well as the geographic location and environmental condition also affect cuticular wax composition (Shepherd and Wynne Griffiths, 2006, Wu et al., 2023).

The fruit of *C. aurantium* L. is in great demand as traditional Chinese medicines, however, the quality of randomly collected *C. aurantium* L. fruit cannot be ensured. In practical agricultural production, a large number of young fallen fruits are widely employed in the market as medicinal herbs without any assurance of their quality. Indeed, the different cultivars, growing locations, ripening stages, and extraction methods can influence the chemical composition of *C. aurantium* L. (Gaff, Esteban‐Decloux, & Giampaoli, 2020). Most of the comparative investigations have only focused on the chemical and biological aspects of different *C. aurantium* L. varieties, including their essential oil constituents and pharmacological actions.

The discrimination in the wax composition of *C. aurantium* L. from different growing locations was rarely explored. In actuality, surface sealing of wax components can efficiently retain lipid soluble components, such as volatile oils, saponins, and certain flavonoids. Herein, we proposed a hypothesis that the geo-authentic area of traditional Chinese herbs has an impact on the type of surface wax of *C. aurantium* L. fruits. Therefore, the objective of the present study was to analyze the surface cuticular wax composition of the cultivated *C. aurantium* fruit peel from different regions by gas chromatography–mass spectrometry (GC–MS).

## 2 Materials and methods

### 2.1 Chemicals

All chemicals used in this work were analytical grade. Chloroform was from General-reagent® (Shanghai, China). BSTFA with 1% TMCS was obtained from TCI (Shanghai, China). Pure helium (99.999%) was obtained from Liufang Industrial Gas Co., Ltd. (Suzhou, China). Normal alkane standards (C-7 to C-40) were purchased from Zhenzhun Biotechnology Co., Ltd. (Shanghai, China).

### 2.2 Plant materials and extraction of cuticular wax

From June to August 2022, *C. aurantium* L. fruits were picked in three traditional production areas: Xingan County in Jiangxi, Yiyang City in Hunan, and Neijiang City in Sichuan. After harvesting, *C. aurantium* L. was dried at 45°Celsius and stored in the natural shade. We obtained two sizes of fruit materials in Jiangxi Province, including normal-sized young fruits (referred to as ‘JX’), and young fruits with diameters<1.0 cm (referred to as ‘JY’). Generally, Jiangxi is recognized as the geo-authentic area for the planting of *C. aurantium* L. trees. The fruit materials from Hunan Province and Sichuan Province were referred to as ‘SC’ and ‘HN’ in this study, respectively.

The fruit peel with a diameter of 1 cm^2^ was cut, and each peel was extracted with 10 mL of chloroform at 60 °C for 5 min. The extraction solution (5 mL) was blown dry with nitrogen. The extract was dissolved in 500 μL of chloroform and 160 μL of BSTFA (with 1% TMCS) was added to derivatize for 1 h at 70 °C. The sample was cooled for 30 min, followed by a centrifugation step at 25 °C (12,000 rpm). 200 μL of the supernatant was used for GC–MS analysis. Quality control (QC) samples were prepared by mixing equal amounts of extracts from all plant samples, and the volume of QC was the same as the plant sample.

### 2.3 GC–MS analysis of *Citrus aurantium* L. fruit peel

An Agilent 7890B gas chromatography system, coupled with an Agilent 5977A MSD system (Agilent Technologies Inc., CA, USA). A DB-5MS capillary column (30 m × 0.25 mm × 0.25 μm, Agilent J & W Scientific, Folsom, CA, USA) was used to separate *C. aurantium* L. fruit with the carrier gas (helium) at a flow rate of 1.0 mL/min. The solvent delay time was set at 5 min. The injector temperature was set at 260 °C, and the injection volume was 1 μL in splitless mode. The initial column oven temperature was 80 °C for 2 min; then increased at a rate of 15 °C/min to 260 °C, and held isothermal for 10 min; finally increased at a rate of 5 °C/min to 315 °C, and then maintained for 10 min. Mass spectrometry parameters were set as follows: ion source temperature, 230 °C; quadrupole temperature, 150 °C; electron impact (EI), 70 eV. Mass spectra were recorded from 50 to 650 *m*/*z* in full scan mode (Jiang et al., 2022). The quantification of the metabolites was performed by external standards (C7-C40 *n*-alkane mixture) with the known content.

Chemical compounds in each sample were identified based on the retention time on the DB-5MS column and matching the National Institute of Standards and Technology (NIST) mass-spectral libraries. In each sample, all peak signal intensities were segmented and normalized according to the internal standards with RSD > 0.3. After normalization, redundancy removal, and peak merging were conducted to obtain the data matrix (Kou et al., 2022).

### 2.4 Statistical analysis

Principle component analysis (PCA) and orthogonal partial least-squares-discriminant analysis (OPLS-DA) were conducted by SIMCA 14.1. One-way analysis of variance (ANOVA) and two-tailed Student's *t*-test were further conducted for difference comparison. After the data was normalized using the function ‘scale’, correlation analysis was performed using the ‘cor’ function and the ‘corolot’ function from the corolot package in R-studio. Statistical data were shown as mean ± standard deviation. P < 0.05 were considered statistically significant.

## 3 Results

### 3.1 Association between chemical profiles of *C. Aurantium* L. fruit peel and growing location

**Fig. 1:**
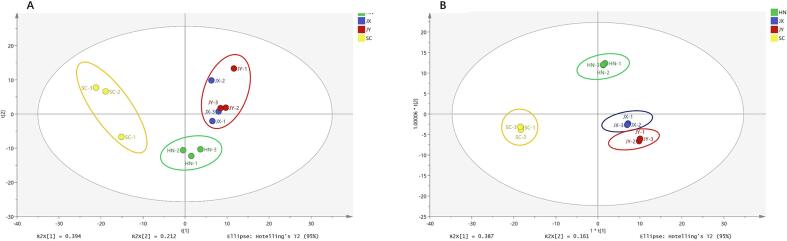
PCA (A) and OPLS-DA (B) scores obtained after modeling the original data matrix of *Citrus aurantium* L. fruit peel from different regions (HN is shown in green, JX in blue, JY in red, and SC in yellow). (For interpretation of the references to color in this figure legend, the reader is referred to the web version of this article.)

**Fig. 2:**
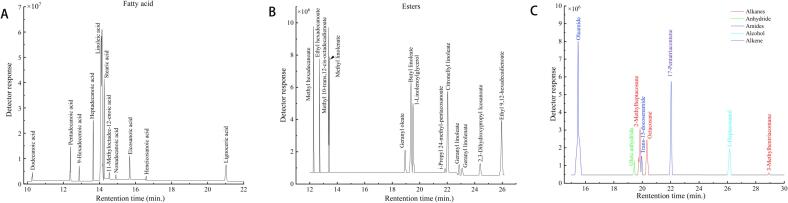
GC–MS chromatogram of the identified cuticular waxes in *Citrus aurantium* L. fruit peel. Total ion chromatogram (TIC) intensity of fatty acid (A); TIC intensity of easters (B); TIC intensity of alkanes, alkene, anhydride, amides, and alcohol (C).

The sensory properties of the fruits were highly variable between cultivars in terms of appearance, color, odor, texture, and taste. The color of dried fruits is one of the most substantial parameters to evaluate the quality of dried samples (Farahmandfar, Tirgarian, Dehghan, & Nemati, 2019). It was observed that HN and SC samples had no white crystallization on the surface of the peels, while the surface of the JY peel appeared whitish, and the JX peel showed apparent whitening. Wax crystal diversity is determined by the proportions of wax components (Chu et al., 2017). Thus, we assume that the varying degree of white crystallization of *C. aurantium* L. from different cultivated areas is due to the cuticular components of the fruit peel. After GC–MS analysis, a total of 349 chemical compositions were detected in *C. aurantium* L. fruit peel. The peak area was used as an indicator for multivariate analysis, such as PCA and OPLS-DA, to elucidate the variation among the *C. aurantium* L. fruit peels from three different growing locations. The peels of *C. aurantium* L. from different regions were clustered into three different groups based on PCA (Fig. 1A) and OPLS-DA (Fig. 1B). The two samples from Jiangxi Province, JX and JY, were gathered in the same group, and the SC sample differed greatly from the other three samples (HN, JX, and JY). Variable importance of projection (VIP) values obtained from the OPLS-DA model were used to rank the overall contribution of each variable to group discrimination. Furthermore, all the metabolites were further analyzed by ANOVA. Therefore, the differential metabolites were selected based on VIP greater than 1.0 from OPLS-DA and p < 0.05 from ANOVA. Finally, a total of 97 molecules were selected for the classification of *C. aurantium* L. from different regions, which included 33 wax components representing 11 fatty acids, 14 esters, 2 amides, 1 anhydride, 3 alkanes, 1 alkene, and 1 alcohol (Fig. 2 and Table 1).

**Table 1:** Identified wax constituents in *C. aurantium* L. fruit peel extract, their retention times (min) and relative concentrations (a relative percentage of the area of peaks, %) in each peel sample (HN, JX, JY, and SC).

Retention times (min)	Waxes	HN (%)	JX (%)	JY (%)	SC (%)
10.283	Dodecanoic acid	0.06 ± 0.00	0.10 ± 0.01	0.12 ± 0.04	0.10 ± 0.01
12.285	Methyl hexadecanoate	0.13 ± 0.03	0.19 ± 0.02	0.25 ± 0.06	0.10 ± 0.04
12.399	Pentadecanoic acid	0.22 ± 0.08	0.40 ± 0.08	0.51 ± 0.15	0.15 ± 0.04
12.723	Ethyl hexadecanoate	0.01 ± 0.00	0.04 ± 0.02	0.31 ± 0.17	0.04 ± 0.03
12.884	9-Hexadecenoic acid	0.15 ± 0.04	0.22 ± 0.01	0.36 ± 0.06	0.14 ± 0.01
13.384	Methyl 10-trans,12-*cis*-octadecadienoate	0.01 ± 0.00	0.03 ± 0.01	0.12 ± 0.05	0.02 ± 0.01
13.424	Methyl linolenate	0.01 ± 0.00	0.02 ± 0.01	0.09 ± 0.04	0.02 ± 0.00
13.654	Heptadecanoic acid	0.56 ± 0.02	0.77 ± 0.01	0.94 ± 0.12	0.32 ± 0.11
14.088	Linoleic acid	1.67 ± 0.09	2.27 ± 0.16	2.65 ± 0.04	1.67 ± 0.34
14.265	Stearic acid	1.21 ± 0.06	1.89 ± 0.17	2.05 ± 0.11	1.04 ± 0.29
14.554	11-Methyloctadec-12-enoic acid	0.02 ± 0.00	0.01 ± 0.00	0.02 ± 0.01	0.00 ± 0.00
14.916	Nonadecanoic acid	0.03 ± 0.00	0.06 ± 0.02	0.07 ± 0.01	0.03 ± 0.01
15.481	Oleamide	0.19 ± 0.02	0.07 ± 0.05	0.21 ± 0.05	0.36 ± 0.05
15.682	Eicosanoic acid	0.24 ± 0.03	0.45 ± 0.10	0.45 ± 0.04	0.25 ± 0.08
16.606	Heneicosanoic acid	0.04 ± 0.00	0.08 ± 0.04	0.08 ± 0.01	0.04 ± 0.01
18.941	Geranyl oleate	0.04 ± 0.01	0.04 ± 0.01	0.09 ± 0.05	0.02 ± 0.01
19.366	Butyl linoleate	0.07 ± 0.01	0.07 ± 0.02	0.20 ± 0.08	0.09 ± 0.04
19.445	Oleic anhydride	0.00 ± 0.00	0.00 ± 0.00	0.01 ± 0.00	0.01 ± 0.00
19.520	1-Linolenoylglycerol	0.15 ± 0.04	0.01 ± 0.00	0.03 ± 0.01	0.05 ± 0.02
19.649	2-Methylheptacosane	0.02 ± 0.00	0.05 ± 0.01	0.03 ± 0.00	0.07 ± 0.01
19.981	Trans-13-docosenamide	0.02 ± 0.00	0.01 ± 0.00	0.00 ± 0.00	0.01 ± 0.00
20.334	Octacosane	0.09 ± 0.03	0.15 ± 0.03	0.22 ± 0.05	0.07 ± 0.02
21.018	Lignoceric acid	0.29 ± 0.03	0.44 ± 0.09	0.62 ± 0.20	0.26 ± 0.08
21.860	i-Propyl 24-methyl-pentacosanoate	0.01 ± 0.00	0.01 ± 0.00	0.02 ± 0.01	0.02 ± 0.01
22.046	Citronellyl linoleate	0.02 ± 0.01	0.04 ± 0.01	0.09 ± 0.04	0.01 ± 0.00
22.071	17-Pentatriacontene	0.00 ± 0.00	0.01 ± 0.00	0.02 ± 0.01	0.00 ± 0.00
22.867	Geranyl linoleate	0.01 ± 0.00	0.02 ± 0.01	0.05 ± 0.02	0.00 ± 0.00
22.907	Geranyl palmitoleate	0.00 ± 0.00	0.01 ± 0.00	0.07 ± 0.04	0.00 ± 0.00
23.094	Geranyl linolenate	0.00 ± 0.00	0.01 ± 0.00	0.04 ± 0.03	0.00 ± 0.00
24.393	2,3-Dihydroxypropyl Icosanoate	0.03 ± 0.01	0.01 ± 0.00	0.06 ± 0.02	0.11 ± 0.04
25.974	Ethyl 9,12-hexadecadienoate	0.02 ± 0.00	0.02 ± 0.00	0.03 ± 0.01	0.01 ± 0.00
26.147	1-Heptacosanol	0.06 ± 0.02	0.17 ± 0.02	0.21 ± 0.09	0.09 ± 0.05
28.942	3-Methylhentriacontane	0.00 ± 0.00	0.00 ± 0.00	0.00 ± 0.00	0.01 ± 0.00

### 3.2 Implication of wax composition in the identification of *C. aurantium* L. from different regions

**Fig. 3:**
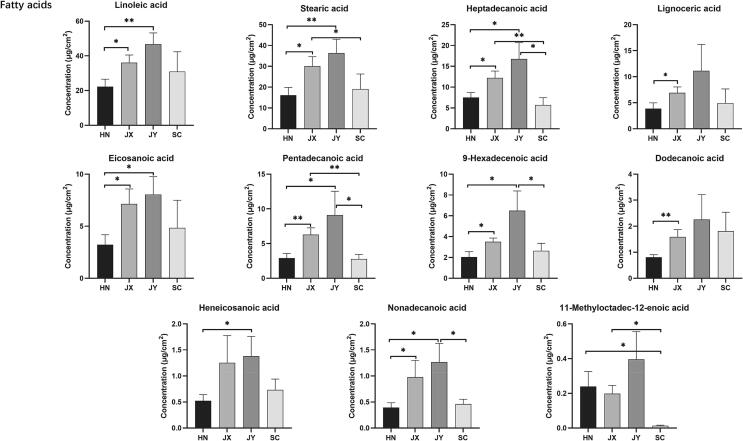
Comparison of fatty acids content in four *C. aurantium* L. fruit peels (HN, JX, JY, and SC; *P < 0.05; **P < 0.01; ***P < 0.001).

The major contributors among wax components to the discrimination of *C. aurantium* L. were identified after statistical analysis. We also quantified the amount of each wax component showing that the predominant compounds were fatty acids, for example, the percentage of linoleic acid and stearic acid in the total content was larger than 1%, followed by heptadecanoic acid, lignoceric acid, eicosanoic acid, pentadecanoic acid, and 9-hexadecenoic acid, exceeding 0.1% (Table 1). Additionally, the amount of saturated fatty acid was higher than that of unsaturated fatty acids (Table S1). There was no essential difference in the fatty acid content between JX and JY peels, but most of the fatty acids were found at significantly higher levels in JX and JY peels, compared to HN and SC peels, with the exception of 11-methyloctadec-12-enoic acid (Fig. 3).

**Fig. 4:**
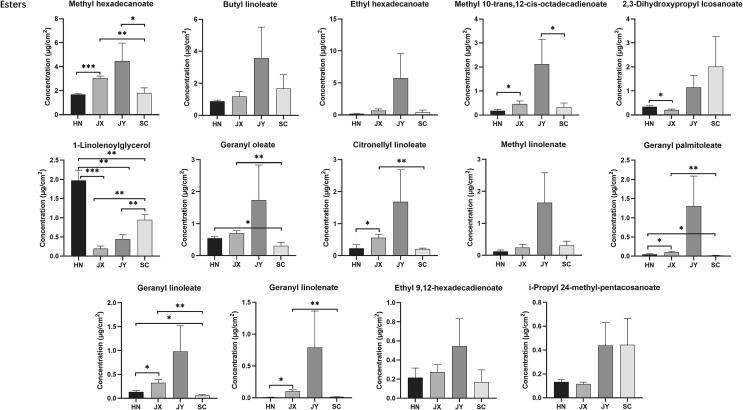
Comparison of esters content in four *C. aurantium* L. fruit peels (HN, JX, JY, and SC; *P < 0.05; **P < 0.01; ***P < 0.001).

Esters were the second most abundant component of the wax compositions, with a lower concentration than fatty acids, aiding in the differentiation of *C. aurantium* L. from different regions. Foremost among these differential esters was methyl hexadecanoate, which was the only one with a total content of more than 0.1%. The majority of other esters were present at trace concentrations. Likewise, geranyl oleate, citronellyl linoleate, geranyl palmitoleate, geranyl linoleate, and geranyl linolenate were present at trace levels in all four *C. aurantium* L. samples, whereas their amounts in HN and SC samples were much lower levels versus JX and JY samples (Fig. 4). Furthermore, it was observed that 1-linolenoylglycerol had a higher content in HN and SC samples than in JX and JY samples. The remaining esters were comparable between the tested groups.

**Fig. 5:**
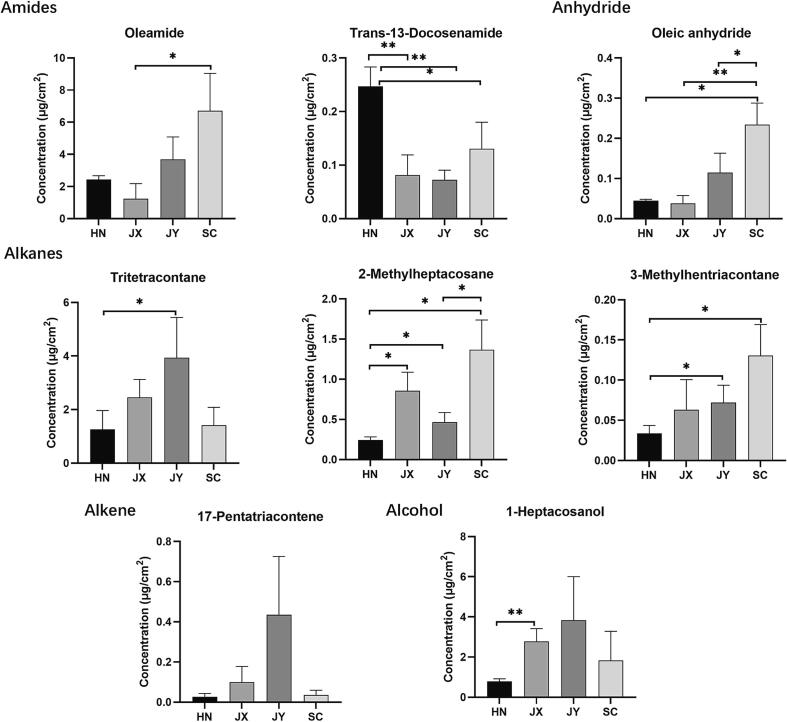
Comparison of 2 amides, 1 anhydride, 3 alkanes, 1 alkene, and 1 alcohol in four *C. aurantium* L. fruit peels (HN, JX, JY, and SC; *P < 0.05; **P < 0.01; ***P < 0.001).

Aliphatic alcohol, alkane, and alkene chemical groups were minor wax components in *C. aurantium* L. samples, which contribute less to the discrimination of *C. aurantium* L. fruits. It was characterized by a significantly higher level of both 2-methylheptacosane and 3-methylhentriacontane from the SC sample than the fruit peel from HN and JX. *C. aurantium* L. peel extracts were also found to contain other waxes such as amide and anhydride in trace amounts (Fig. 5).

### 3.3 Correlation analysis of different chemical categories of wax components

**Fig. 6:**
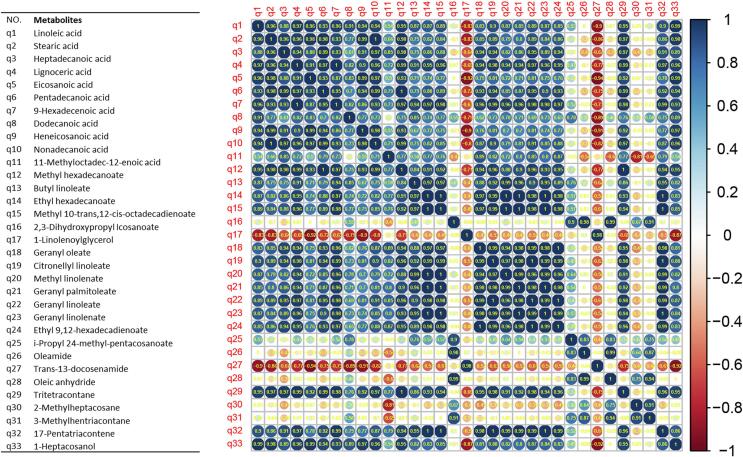
Correlation analysis of all waxes responsible for the differences in four *C. aurantium* L. fruit peels.

The waxes responsible for the differences of *C. aurantium* L. fruit peel from different regions were subjected for correlation analysis, shown in Fig. 6. There was a strong positive correlation between long-chain fatty acids (q1-11), except for q8 (dodecanoic acid) and q11 (11-methyloctadec-12-enoic acid), which were poorly correlated with the other long-chain fatty acids. Strong positive correlations were found between the esters q12-25, excluding q16, 17 and 25, where the correlation was weaker for q16 (2,3-dihydroxypropyl lcosanoate) and q25 (i-propyl 24-methyl-pentacosanoate). Whereas q17 (1-linolenoylglycerol) showed negative correlation with most of the long-chain fatty acids and esters. Two amides (q26-27, oleamide and *trans*-13-docosenamide), one anhydride (q28, oleic anhydride), and two alkanes (q30-31, 2-methylheptacosane and 3-methylhentriacontane) had negative correlations with long-chain fatty acids and esters. It was observed that alkane (q29, tritetracontane), alkene (q32, 17-pentatriacontane), and alcohol (q33, 11-heptacosanol) showed strong positive correlations with long-chain fatty acids and esters.

Overall, the majority of wax compositions in the *C. aurantium* L. fruit peels from HN and SC showed significantly lower amounts in JX and JY peel after excluding trace components. The data obtained from the analysis of *C. aurantium* L. peel indicated that the high presence of fatty acids was considered to be the main determinants in the identification of *C. aurantium* L. from different growing locations. Furthermore, a combination of two or more compositions with similar correlations could also be an indicator for the identification of *C. aurantium* L. from different growing locations.

## 4 Discussion

*C. aurantium* L. is one of the most important Chinses medicinal plants, which is a geo-authentic herb cultivated in Jiangxi Province. In order to know more details on the difference in *C. aurantium* L. fruit peels from different regions, the present work focused on the use of GC–MS technology to achieve a comprehensive analysis of the *C. aurantium* L. fruit peel. The results showed that *C. aurantium* L. peel extract had a complex chemical profile, containing a large number of constituents with contents lower than 0.1%, which could be affected by current chromatographic condition. JX and JY samples yielded the highest percentage of fatty acids such as heptadecanoic acid and lignoceric acid. This abundance of cuticular waxes was appropriate for diversity analysis mainly reflecting differences in cultivars, which might in turn cause differences in the quality of traditional herbal plants. Further studies could also be carried out with respect to the functional characterization and metabolic pathways of fatty acids.

It has been found in previous studies that linoleic acid, palmitic acid, and oleic acid are the major fatty acids in *C. aurantium* L. lipid composition (Benayad et al., 2021, Waheed et al., 2009). Our study showed that linoleic acid produced higher levels in JX and JY samples compared to HN and SC samples. Although palmitic acid (4.4–5.3%) and oleic acid (0.05–1.85%) were rich in *C. aurantium* L. peel, these two compounds did not contribute to the discrimination of *C. aurantium* L. samples after multivariate analysis. Limonene was the abundance of essential oil component in the *C. aurantium* L. fruit peel serving as a flavoring agent (Azimi and Fatemi, 2016, Boussaada et al., 2007, Farag et al., 2020). Whereas limonene was not present in *C. aurantium* L. peel in our study. Considering the physicochemical properties of wax compositions, hydrophobic solvents are generally used for the extraction of wax components, for example, chloroform, hexane, or petroleum ether (Klavins & Klavins, 2020). To further discriminate and categorize *C. aurantium* L. from different regions, the way forward could be towards developing a new extraction method for obtaining more wax components. We also observed that an essential oil, nerolidol 2 (d34; Fig. S1, Supplementary material), was positively correlated with fatty acids, which contribute to the discrimination of *C. aurantium* L. from different cultivation areas.

It has been shown that the composition of waxes includes long-chain aliphatic components, whereby phytosterols and flavonoids are also part of cuticular wax (Klavins and Klavins, 2020, Shepherd and Wynne Griffiths, 2006). Nonetheless, we screened only three sterols in *C. aurantium* L. after multivariate analysis including campesterol (0.38–0.58%), 24-epicampesterol (0.29–0.41%), and β-sitosterol (1.58–2.74%). The content of these three sterols did not significantly differ between the mentioned groups (HN, JX, JY, and SC). According to the correlation analysis of the significant metabolites in *C. aurantium* L. samples, the sterols (d41-43; Fig. S1) showed positive correlation with fatty acids. Moreover, the multivariate analysis also selected the two most abundant flavonoids, tangeretin and nobiletin, which were responsible for the difference in *C. aurantium* L. from different regions. The highest relative content of tangeretin was found in HN (4.01%), JX (6.10%), JY (4.40%) samples, and also higher in SC sample (2.52%). Nobiletin was the higher amount in HN (1.18), JX ((2.82), JY (1.27), and highest in SC (6.45). There were positive correlations between tangeretin (d37; Fig. S1) and fatty acids, while nobiletin (d40; Fig. S1) showed negative correlation with fatty acids. We assume that flavonoids could also be identifiers due to the high content and significant correlation with fatty acids in *C. aurantium* L. from different regions.

In the present study, molecular marker analysis was used to investigate the association between morphological and chemical variations of the fruit peels in three growing areas. It appears that the wax content of the *C. aurantium* L. varied depending on the growing location with the highest levels of fatty acids found in JX and JY samples. Herein, not much can be concluded for sure on the influence of the location in which *C. aurantium* L. trees are grown on the cuticular wax composition. The observed variation may be attributed to the nature of the soil, as well as to the geographical and climatic conditions.

## 5 Conclusions

There was no significant difference in the fatty acid content between *C. aurantium* L. fruit peels collected from the same geo-authentic area (Jiangxi, samples: JX & JY), but most of the fatty acids showed significantly higher levels in JX and JY fruit peels compared to those from other regions (Hunan and Sichuan, samples: HN and SC). In addition, esters were of little importance in distinguishing *C. aurantium* L. from different regions due to their low contents. Although the amounts of some esters, such as geranyl oleate, citronellyl linoleate, geranyl palmitoleate, geranyl linoleate, and geranyl linolenate, were much higher levels in JX and JY samples than in HN and SC samples. In short, the majority of wax composition in *C. aurantium* L. fruit peel from Jiangxi, the geo-authentic area of *C. aurantium* L., showed significantly higher amounts compared to Sichuan and Hunan after excluding trace components. Hence, the findings indicated that the high presence of fatty acids possibly contributes principally to the discrimination of the quality of *C. aurantium* L. from different growing locations.

## CRediT authorship contribution statement

**Yan Li:** Data curation, Formal analysis, Methodology, Investigation, Validation. **Jie Liu:** Data curation, Formal analysis, Methodology, Investigation, Validation. **Jie Li:** Formal analysis, Investigation. **Haijing Xiao:** Formal analysis, Investigation. **Yiyun Xu:** Formal analysis, Investigation. **Siqing Fan:** Resources, Investigation. **Zhaoqi Xie:** Resources, Investigation. **Min Guo:** Resources, Investigation. **Jiaxin Yang:** Resources, Investigation. **Xue Jing:** Formal analysis, Investigation. **Chunsong Cheng:** Conceptualization, Supervision, Project administration, Methodology, Supervision, Validation.

## Declaration of Competing Interest

The authors declare that they have no known competing financial interests or personal relationships that could have appeared to influence the work reported in this paper.

## Data Availability

Data will be made available on request.
