# Supplementary material for: Chemical characterization and discovery of novel quality markers in Citrus aurantium L. fruit from traditional cultivation areas in China using GC–MS-based cuticular waxes analysis
Source: Food Chem X. 2023 Sep 20;20:100890. doi: 10.1016/j.fochx.2023.100890 (PMC10740023; doi:10.1016/j.fochx.2023.100890)
Supplement: Supplementary data 1 [file mmc1.docx]

**Chemical characterization and discovery of novel quality markers** **in *Citrus aurantium* L. fruit from traditional cultivation areas in China using GC-MS-based cuticular waxes analysis**

**Figure S1**. Correlation analysis of all waxes, essential oil, flavonoids, and sterols responsible for the differences of four *C. aurantium* L. fruit peels.


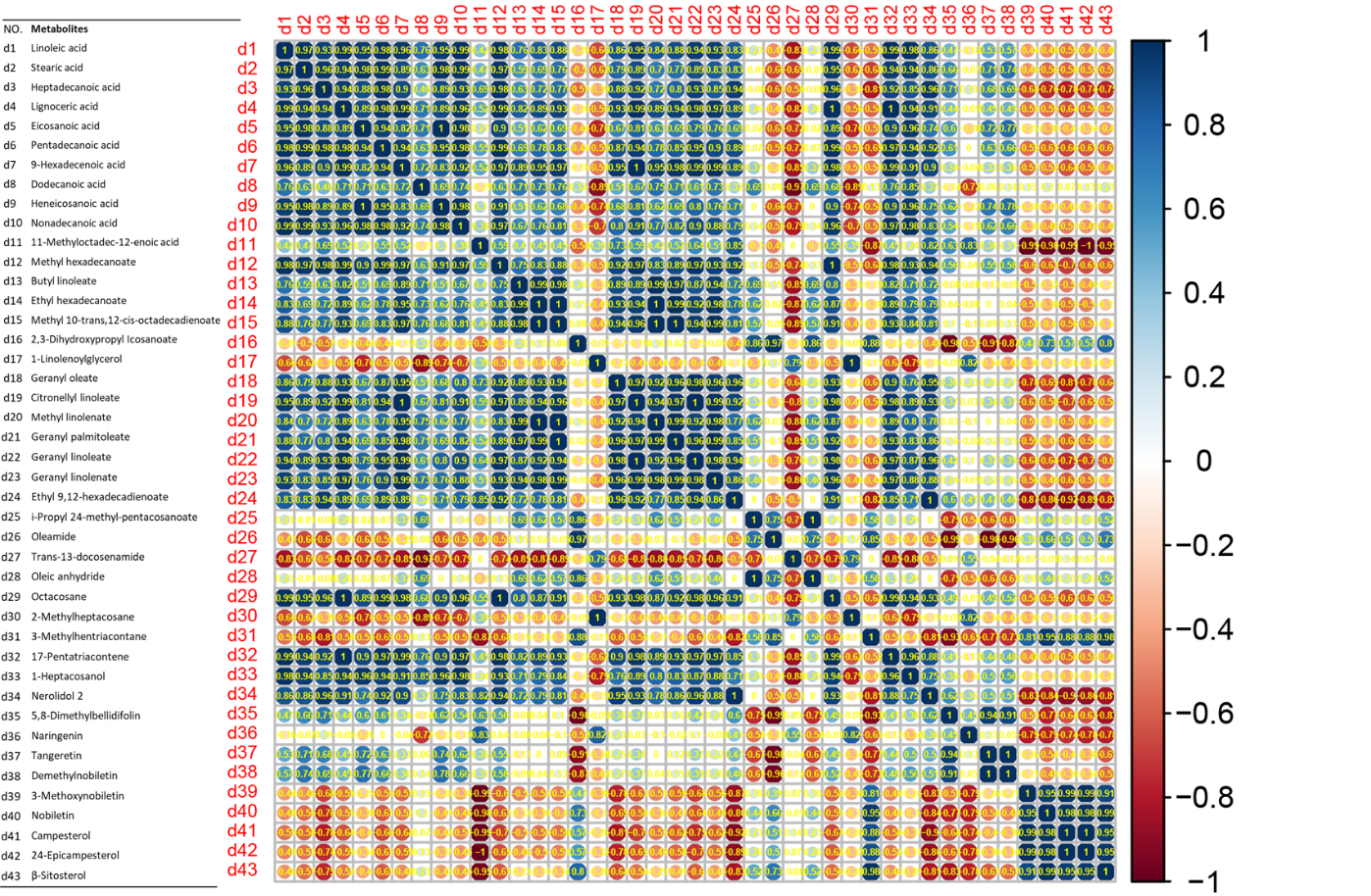


**Table S1.** The content of cuticular waxes in *C. aurantium* L. (HN, JX, JY, and SC) from different regions.

| **Metabolites** | **HN** | **JX** | **JY** | **SC** |
| --- | --- | --- | --- | --- |
| Linoleic acid | 22.28 ± 4.29 | 36.1 ± 4.47 | 46.7 ± 6.46 | 30.94 ± 11.43 |
| Stearic acid | 16.12 ± 3.73 | 30.1 ± 4.56 | 36.4 ± 6.61 | 19.14 ± 7.16 |
| Heptadecanoic acid | 7.48 ± 1.24 | 12.2 ± 1.63 | 16.8 ± 3.88 | 5.72 ± 1.74 |
| Lignoceric acid | 3.87 ± 1.11 | 6.93 ± 1.11 | 11.2 ± 5.04 | 4.93 ± 2.72 |
| Eicosanoic acid | 3.22 ± 0.96 | 7.14 ± 1.43 | 8.06 ± 1.7 | 4.84 ± 2.65 |
| Pentadecanoic acid | 2.89 ± 0.69 | 6.29 ± 0.98 | 9.1± 3.44 | 2.77 ± 0.65 |
| 9-Hexadecenoic acid | 2.03 ± 0.52 | 3.51 ± 0.35 | 6.5 ± 1.89 | 2.63 ± 0.74 |
| Dodecanoic acid | 0.81 ± 0.10 | 1.59 ± 0.28 | 2.26 ± 0.96 | 1.81 ± 0.73 |
| Heneicosanoic acid | 0.52 ± 0.12 | 1.25 ± 0.52 | 1.38 ± 0.38 | 0.73 ± 0.21 |
| Nonadecanoic acid | 0.39 ± 0.09 | 0.98 ± 0.32 | 1.27 ± 0.36 | 0.46 ± 0.09 |
| 11-Methyloctadec-12-enoic acid | 0.24 ± 0.09 | 0.2 ± 0.05 | 0.4 ± 0.16 | 0.01 ± 0.004 |
| Methyl hexadecanoate | 1.68 ± 0.1 | 3.06 ± 0.17 | 4.46 ± 1.51 | 1.8 ± 0.44 |
| Butyl linoleate | 0.87 ± 0.07 | 1.17 ± 0.3 | 3.58 ± 1.93 | 1.67 ± 0.87 |
| Ethyl hexadecanoate | 0.14 ± 0.03 | 0.65 ± 0.27 | 5.76 ± 3.83 | 0.39 ± 0.35 |
| Methyl 10-trans,12-cis-octadecadienoate | 0.17 ± 0.06 | 0.45 ± 0.13 | 2.11 ± 1.02 | 0.32 ± 0.18 |
| 2,3-Dihydroxypropyl Icosanoate | 0.33 ± 0.05 | 0.2 ± 0.03 | 1.14 ± 0.51 | 2.02 ± 1.25 |
| 1-Linolenoylglycerol | 1.97 ± 0.27 | 0.19 ± 0.07 | 0.45 ± 0.11 | 0.95 ± 0.14 |
| Geranyl oleate | 0.55 ± 0.05 | 0.7 ± 0.08 | 1.73 ± 1.11 | 0.3 ± 0.10 |
| Citronellyl linoleate | 0.23 ± 0.11 | 0.56 ± 0.11 | 1.68 ± 1.01 | 0.21 ± 0.03 |
| Methyl linolenate | 0.12 ± 0.04 | 0.24 ± 0.1 | 1.64 ± 0.94 | 0.31 ± 0.13 |
| Geranyl palmitoleate | 0.04 ± 0.02 | 0.1 ± 0.02 | 1.3 ± 0.78 | 0.02 ± 0.005 |
| Geranyl linoleate | 0.13 ± 0.03 | 0.32 ± 0.07 | 0.98 ± 0.54 | 0.06 ± 0.02 |
| Geranyl linolenate | 0.01 ± 0.001 | 0.1 ± 0.02 | 0.79 ± 0.57 | 0.01 ± 0.001 |
| Ethyl 9,12-hexadecadienoate | 0.22 ± 0.10 | 0.27 ± 0.08 | 0.55 ± 0.29 | 0.17 ± 0.13 |
| i-Propyl 24-methyl-pentacosanoate | 0.13 ± 0.02 | 0.12 ± 0.02 | 0.44 ± 0.19 | 0.44 ± 0.22 |
| Oleamide | 2.44 ± 0.23 | 1.23 ± 0.95 | 3.69 ± 1.39 | 6.71 ± 2.33 |
| Trans-13-docosenamide | 0.25 ± 0.04 | 0.08 ± 0.04 | 0.07 ± 0.02 | 0.13 ± 0.05 |
| Oleic anhydride | 0.05 ± 0.003 | 0.04 ± 0.02 | 0.11 ± 0.05 | 0.23 ± 0.05 |
| Octacosane | 1.26 ± 0.70 | 2.46 ± 0.67 | 3.93 ± 1.5 | 1.41 ± 0.67 |
| 2-Methylheptacosane | 0.24 ± 0.04 | 0.86 ± 0.23 | 0.47 ± 0.12 | 1.37 ± 0.37 |
| 3-Methylhentriacontane | 0.03 ± 0.01 | 0.06 ± 0.04 | 0.07 ± 0.02 | 0.13 ± 0.04 |
| 17-Pentatriacontene | 0.03 ± 0.02 | 0.1 ± 0.08 | 0.44 ± 0.29 | 0.04 ± 0.02 |
| 1-Heptacosanol | 0.79 ± 0.13 | 2.78 ± 0.64 | 3.83 ± 2.17 | 1.83 ± 1.45 |
